# Towards identifying the characteristics of youth with severe and enduring mental health problems in practice: a qualitative study

**DOI:** 10.1007/s00787-023-02325-2

**Published:** 2023-12-26

**Authors:** C. H. Bansema, R. R. J. M. Vermeiren, L. Nijland, R. de Soet, J. Roeleveld, H. van Ewijk, L. A. Nooteboom

**Affiliations:** 1 LUMC Curium-Department of Child and Adolescent Psychiatry, Leiden University Medical Center, Post Box 15, 2300 AA Leiden, The Netherlands; 2 Youz, Parnassia Group, The Hague, The Netherlands

**Keywords:** Severity, Long term nature of mental health problems, Interrelated mental health problems, Child and adolescent psychiatry, Qualitative research

## Abstract

A group of youth with severe and enduring mental health problems (SEMHP) falls between the cracks of the child-and-adolescent psychiatry (CAP) system. An insufficient understanding of these youth’s mental health problems results in a failure to accurately identify and provide support to these youth. To gain a deeper understanding, the aim of this study is to explore characteristics of youth with SEMHP in clinical practice based on the experiences of youth and clinicians in CAP. This qualitative study consisted of 20 semi-structured interviews with 10 youth with lived experience and 10 specialized clinicians in CAP. Both a thematic and content analysis was conducted to identify, assess, and report themes associated with youth with SEMHP. Themes were individual characteristics such as trauma, masking, self-destructive behavior, interpersonal distrust as well as environmental and systematic characteristics including parental stressors, social isolation and societal stressors, which go beyond the existing classifications. These characteristics profoundly impact youth’s daily functioning across various life domains, creating an interactive process, ultimately leading to elusive mental health problems and overwhelming feelings of hopelessness. The authors recommend proper assessment of characteristics in all life domains affected and their perpetuating effect on SEMHP during diagnostics in CAP. Engaging in a dialogue with youth themselves is crucial due to the nature of youth’s characteristics, which frequently transcend traditional classifications and may not be immediately discernible. It also requires an integrated care approach, entailing collaborations between educational institutions and mental healthcare providers, and attention to potential indicators of deficits in the healthcare system and society.

**Supplementary Information:**

The online version contains supplementary material available at 10.1007/s00787-023-02325-2.

## Introduction

There is a small group of youth (aged 16–25 years) with severe and enduring mental health problems (SEMHP) who appear to be systematically stuck in the current child and adolescent psychiatry (CAP) system [1, 2]. Clinicians perceive these youth (and emerging adults, also referred to as youth in this paper), as complex due to the multiple and (often) interrelating mental health problems [1–3]. Recent evidence suggests an increase in the incidence of complex mental health problems including self-harm [3]. However, fundamental knowledge about the mental health problems faced by these youth, and the reasons explaining their growing complexity, remains limited [4]. Therefore, a deeper understanding of their characteristics is needed, to improve timely recognition of and adequate help for these youth [4].

Over the last decades, the Diagnostic and Statistical Manual (DSM) has become the norm for understanding mental health problems, including SEMHP. Globally, DSM classifications have turned into the leading language in psychiatry. Over time, criticism of using the DSM criteria in practice has increased substantially [5, 6]. The allure of the existing evidence base, based on DSM classifications, restricts us from providing holistic care and understanding individuals as a ‘whole’. As a result, youth with SEMHP regularly fall between the cracks of the mental health system; their problems often do not fit a specific classification or fit multiple classifications over time. This leads to undetected mental health problems, misdiagnoses, as well as overdiagnoses [7, 8], and unmet needs for youth with SEMHP.

In addition, the misdiagnoses of SEMHP may be partly explained by the current mental health care’ focus on determining the severity of disorders based on the number of symptoms a person is experiencing. In previous editions of the DSM, the severity was measured on symptom rating scales such as GAF scores [9, 10]. Although the current DSM (DSM-5) has abandoned this practice [11], the emphasis is still on evaluating the severity of specific symptoms related to a disorder [10].

By focusing on specific symptoms rather than its entirety, the interaction and interrelatedness of underlying vulnerability and mental health problems is overlooked. Also, the characteristics outside these symptom-defined disorders that affect youth’s daily functioning such as resilience, social support, or cultural and societal expectations are missed [10]. In addition, growing concerns arise over youth with SEMHP who are dealing with multiple societal stressors such as the COVID-19 effects, pressure from social media, and stress about the future (climate changes, housing, livelihood security) [12]. It is, therefore, essential, in these times of change and uncertainty, to explore the expression of SEMHP and determine the associated characteristics with SEMHP that are not only limited to personal factors, but also concern societal stressors they have to deal with.

Unfortunately, studies into the characteristics of youth with SEMHP are sparse. A prior study identified multiple, co-occurring, interrelated mental health problems and trauma-related stressors associated with these youth [2]. According to this study, the complex presentations can lead to difficulties in accessing appropriate services, inadequate treatment outcomes, and high rates of hospitalization and involvement with the criminal justice system. Moreover, the few studies into youth with SEMHP are mostly quantitatively data-driven, which has the advantage of exploring the relation between different mental health problems, but is lacking potential explanations or context-dependent nuances [2, 13, 14].

To better understand the needs of youth with SEMHP, in the complex contexts these youth find themselves, a more in-depth approach with a focus on explaining the expression of their mental health problems is needed [15]. Therefore, this qualitative study aims to explore the characteristics of youth with SEMHP by conducting semi-structured interviews with youth with lived experience and clinicians specialized in child and adolescent psychiatry [16, 17]. This qualitative method, including the perspectives of youth and clinicians [16], fits for gaining a deeper understanding of the characteristics of these youth. It is highly relevant to engage the perspectives of youth and clinicians in research, since both perspectives contribute to a better understanding of clinical practice. However, it is noteworthy that youth’s and clinicians' conceptualization of mental health problems received relatively little attention in current research [18].

## Method

### Setting and study design

This study is part of ‘DevelopRoad’, a research project with the objective to gain a better understanding of the characteristics and needs of youth with SEMHP, focused on CAP facilities in the Netherlands. The project team consists of researchers, peer workers, and experts in the field, associated with LUMC Curium, a CAP facility in the Netherlands. The Medical Ethics Review Board of Leiden University Medical Center concluded that the research project was not subject to the Medical Research Involving Human Subject Act (WMO) and complied with the Netherlands Code of Conduct for Research Integrity (reference number: N21.094).

The overall research project is explorative, following an inductive grounded theory approach [19]. In doing so, we go through an iterative cycle of data collection, analysis and reflection to explore characteristics of youth with SEMHP. For this study, semi-structured interviews were expected to provide sufficient structure to deepen the various topics, while leaving room for the respondents to delve further into topics deemed essential to them. The explorative nature of the interviews enabled us to get an initial idea of the view of youth and clinicians about severe and enduring mental health problems [20]. The Consolidated criteria for Reporting Qualitative Research checklist (Appendix A) was operated to provide an accurate representation of the study carried out [21].

### Participants

In this study, we included 20 participants, including youth with lived experience (*n = *10) and clinicians of CAP facilities (*n = *10). In order to select eligible participants, we have sampled purposively, a non-probability sampling method based on the judgements of the researchers [22]. Participants were included until data saturation was reached. This is the point where no new information emerged and, therefore, no supplemental interviews were needed [20]. We described SEMHP as interrelated and enduring mental health problems that necessitate care, with often loss of all or part of youth’s hope for a better future. Participants for the interviews were eligible according to the following criteria:

#### Youth—informants with lived experience

Youth: (i) between the ages of 16 and 25 years; (ii) who participate in a youth council commission (Dutch National Youth Council (NJR)) or work as an expert by experience (Experienced Experts (ExpEx)); and (iii) who recognize themselves in the description: youth with severe and enduring mental health problems. We included youth with lived experience because of their knowledge about the target group, their ability to reflect, and their experience in sharing their stories [23]. Youth with lived experience were recruited from the NJR and ExpEx and by approaching the contacts of the project peer worker.

#### Professionals—specialized clinicians

Clinicians: (i) affiliated with a CAP facility; (ii) who work with youth with severe and enduring mental health problems; and (iii) who are specialists with final responsibility for treatment. Clinicians were recruited from four CAP facilities in the Netherlands (LUMC Curium, Levvel, Karakter, and Accare).

### Procedure

Participants were informed about the research project through information letters sent by e-mail by one of the two researchers (RS or CB), including a project description, the interviewing process and an informed consent. A youth representative (JR) supported the researchers in formulating the content to ensure youth understood the information. Subsequently, participants were contacted by e-mail or phone. After the participants agreed to participate, they gave written informed consent before the interview. The aims, objectives, voluntary nature of participation, confidentially, and anonymity of the data were discussed verbally and in writing. All participants were offered a 25-euro voucher for their participation. None of the participants refused or dropped out. The participants were assigned a study number to guarantee anonymity.

### Data collection

A pre-prepared topic list guided the interviews (Appendix B). The topic list contained open-ended questions based on an internal focus group with youth and clinicians (2019) and current literature on SEMHP [24–27]. The topic list was modified through a reflexive meeting with all authors. Subsequently, the topic list was tested with a youth representative (JR). The topic list included questions regarding the meaning of severe and enduring mental health problems; and how one (clinicians and the youth themselves) would characterize them. The interviews were conducted between March 2021 and June 2021 by two researchers (RS and CB, both female). The interviews were performed using a digital platform due to the COVID-19 pandemic (Microsoft Teams, Microsoft 365), and lasted between 45 and 60 min. Reflexive meetings to evaluate the interview process and discuss new insights between the two researchers (RS and CB) took place after each interview. Each interview was audio-recorded and transcribed (verbatim) afterward. Field notes were taken during the interviews. The transcripts were saved in a secured digital environment of Leiden University Medical Center. The transcripts were not returned to the participants for comments and correction. Three researchers translated the quotes from Dutch to English (CB, LAN, LIN). Due to the verbatim transcription, the quotes presented in our results section contain literal wordings and might lack fluency.

### Analysis

All transcripts were imported into a software system (Atlas.ti.9). We conducted a thematic analysis to identify, assess, and report the themes within the data [28], and a content analysis to quantify and examine the frequency of the themes [29]. A thematic analysis was conducted following the step-by-step plan of Braun and Clarke [28]. This plan addresses six stages: (1) becoming familiar with the data, (2) generating codes in the data, (3) generating themes, (4) reviewing themes, (5) defining and naming themes, and (6) locating exemplars [28]. A content analysis allows researchers to quantify the data: basic content analyses are approaches using e.g. word counts to analyze the data [30]. In analyzing the transcripts, we applied inductive and deductive strategies [31, 32]. A coding tree was deductively developed based on the existing literature on SEMHP [24–27], supplemented with inductive codes that arose from line-by-line open coding (Appendix C). The first five interviews were coded separately by two researchers (CB and RS) and discussed afterwards to overcome interpretation bias. Differences in coding were resolved by the researchers (CB and RS). After coding approximately 15 out of the 20 interviews (alternating youth and clinicians as much as possible), no additional codes were added, indicating inductive thematic saturation was reached [33]. Subsequently, axial coding took place through further analysis and merger of the coded fragments [34]. During reflexive meetings, two researchers (LAN and CB) discussed the interpretation of the coded fragments.

## Results

### Participants

Youth with lived experience (female *n = *7, male *n = *3) had a mean age of 21 years old (age range in years 19–24). Their self-reported classifications were a combination of depression, personality disorder, eating disorder, autism spectrum disorder, and anxiety disorder. Additional mental health problems were suicidality and impaired functioning in multiple areas of life. Youth with lived experience mentioned a duration of mental health problems around 10–11 years and a duration of receiving in mental health care around 6–7 years. The specialized clinicians (female *n = *5, male *n = *5) had a mean age of 45 years old (age range in years 35–57). These specialized clinicians consisted of child and adolescent psychiatrist (*n = *9) and a child and adolescent psychiatry case manager/psychologist (*n = *1), with a variety of 6–26 years of experience in the CAP setting.

### Descriptions of the terms: enduring and severe

To understand the meaning of SEMHP, we first asked participants to describe the terms *enduring* and *severe* regarding mental health problems*.* According to the participants, enduring was related to (a) the duration of mental health problems, (b) the duration of care, (c) the recurrence of problems, and (d) the invisibility of problems. Severe was associated with (a) hampered functioning in various life domains, (b) multiple classifications, (c) trauma, (d) high-risk behavior, (e) hospitalization, and (f) a high burden. Additional information including the frequency of descriptions can be found in Appendix D.

### Characteristics of youth with SEMHP

The results of the thematic analysis were divided into the following categories (a) the individual context, (b) the family context, (c) the peer context, (d) the societal context, and (e) the impact on daily life. Detailed information about the frequency of the themes per context can be found in Appendix E.

#### Category 1: Individual context

This category describes characteristics of youth with SEMHP related to the individual context, including (a) (childhood) trauma, (b) genetic vulnerability of SEMHP, (c) the role of puberty, (d) masking of the mental health problems, (e) high-risk behavior of youth with SEMHP, and (f) interpersonal distrust in youth with SEMHP.

##### 1.a Trauma

Most participants emphasized the presence of trauma in youth with SEMHP. It was described as an emotional response to experiences like abuse, mostly during childhood. As much as trauma is about the individual, one clinician, and most youth emphasized the importance of the environment in relation to trauma. The clinician described a lack of parental success in dealing with a childhood trauma. Youth mentioned the effect of growing up in an unsafe environment with mistreatment and abuse.

##### 1.b Genetic vulnerability of SEMHP

Most clinicians described an underlying genetic vulnerability for coping with stress, intense emotions, developmental problems, anxiety and mood symptoms, and psychotic symptoms. Youth did not mention genetic vulnerability.

##### 1.c Puberty

Several aspects of puberty were described by the participants, namely (a) experiencing strong emotions; (b) comparing yourself with others; and (c) separation of caregivers, while bearing responsibility can be complicated. In contrast, some participants mentioned that puberty can also be a period in which some problems, such as social anxiety or emotional problems, may diminish for a while and then appear again. The aspect of experiencing event related emotions may affect the presence of the problems. For example, falling in love may contribute to diminishing or masking mental health problems, while a broken relationship can actually aggravate them again.

*“When I had problems in youth mental health care, there was also a phase when I was in puberty. And then, I found out by myself which problems remained and which problems disappeared.”* Youth 3

##### 1.d Masking of mental health problems

Half of the youth described masking their mental health problems for the people in their environment out of shame or to avoid worrying them. In addition, a few clinicians mentioned the masking behavior of youth in treatment.

*“It is much worse what it does to your caregivers or friends than it does to you."* Youth 1

##### 1.e Self-destructive/high-risk behavior of youth with SEMHP

Participants described high-risk behavior in relation to SEMHP, including severe self-mutilation, suicidality, aggression, and substance abuse. Some youth described harming themselves to feel something or to let go of tension. On the other hand, a few clinicians mentioned less visible high risk-behavior, such as youth who gets nowhere and sits at home, which may be a danger to their development and, therefore, a threat to themselves.

*“You don’t know what they avoid and why they avoid; you only see that they avoid. In my view, suicidality is also avoidance to face certain things.”* Clinician 2

##### 1.f Interpersonal distrust in youth with SEMHP

Both youth and clinicians mentioned a very low sense of confidence to tackle obstacles in daily life and a fear of rejection in these youth, whereby youth are afraid of not meeting the expectations of others and themselves. Also, clinicians mentioned a pitfall for caregivers. Caregivers who take over youth’s problems can create a self-defeating side, namely the feeling that everyone is doing everything for the young person who apparently cannot do it himself, resulting in interpersonal distrust.

*"Often youth have a very low confidence in themselves to tackle things. So you can imagine that they will think: I am not at all capable of meeting the demands you are making of me now.”* Clinician 6

#### Category 2: Family context

This category describes characteristics of youth with SEMHP related to the family context, including (a) parental stressors.

##### 2.a Youth with stressed caregivers

Over half of the participants described the presence of parental stressors as a perpetuating factor in youth’s SEMHP. Several factors for a stressed-out family system were mentioned, including caregivers with psychiatric problems, parental financial stress, and disturbed communication. Some participants described parental psychiatric problems, such as addiction problems, developmental problems, and avoidant personality traits contributing to the continuation and maintenance of youth’s SEMHP. Moreover, a few clinicians mentioned parental financial problems such as unemployment impacting youth’s SEMHP. Lastly, clinicians mentioned disturbed communication patterns in the family system, because of the problems youth are dealing with, because of the personal problems of caregivers, or a combination of these two.

*"My caregivers also have a bag of mental health history, so when they judge behavior on what is healthy and what is not healthy, they do it from their point of view. And their point of view is damaged too."* Youth 10

#### Category 3: Peer context

This category describes characteristics of youth with SEMHP related to their peers, including (a) the lack of social support by peers, (b) isolation, and (c) invisibility of SEMHP.

##### 3.a Lack of social support by peers

A lack of social support was mentioned by most participants and explained by youth as (i) a lack of qualitative social relationships; and (ii) being bullied; (iii) the negative reactions of their environment.

##### 3.b Isolation

Participants mentioned isolation of youth with SEMHP and explained this by (i) youth’s feeling that they do not belong to others; (ii) lack of social support by family members and peers. It was explained by the participants as a series of events and behavior such as youth that stop to attend social events, withdraw, drop out of school and eventually disappear from their social environment and become isolated.

##### 3.c Invisibility of SEMHP for peers

Participants mentioned the invisibility of SEMHP for their social environment, including becoming invisible for peers, friends or teachers. For example, peers and teachers at school usually notice the acute absenteeism, however they do not always see the run-up of the problems a youth is experiencing. Participants described that too often the quiet youth with internalized problems are left unnoticed. While the externalized behavior is more noticeable for the social environment, but often misinterpreted.

#### Category 4: Societal context

This category describes characteristics of youth with SEMHP related to the mental health care system and society, including (a) overdiagnoses of multiple classifications by clinicians, (b) hospitalization, (c) societal stigma, and (d) societal stress.

##### 4.a Multiple classifications

Participants mentioned the presence of multiple classifications for youth with SEMHP. Both clinicians and youth explained that this number of classifications existed, because all problems are classified separately. For example, depressive feelings are classified as a depression, quitting eating is classified as an eating disorder, and feeling anxious is classified as an anxiety disorder, while the connection between these problems is not described. As a result, participants mentioned a mismatch in treatment, and inappropriate classifications were not removed from youth’s files. Both clinicians and youth mentioned that this way of describing problems is old-fashioned, and too focused on classifications without seeing the interrelatedness of problems.

##### 4.b Hospitalization of youth with SEMHP

Hospitalization was described as the effect of being hospitalized for a long time, and related to youth with SEMHP as (i) becoming too much accustomed to life in a CAP facility; (ii) experiencing fear of returning to society (consciously or unconsciously); (iii) feeling unprepared to deal with society; and (iv) having a social network consisting of only peers with problems.

*"Also, when you are in psychiatry, long-term admission is not desirable, and in admission, many behaviors of others are adopted. Suppose the problem is already severe; you don’t want it to worsen. So if you become isolated, have no friends, are not safe at home, and have physical complaints, that makes it even more difficult.”* Youth 3

##### 4.c Societal stigma

The presence of societal stigma on SEMHP was mentioned by a few participants. Youth described societal stigma as the feeling that people keep their distance, when youth are trying to be open about their problems. According to these youth, people are often distanced because of a lack of knowledge about these problems in society. Participants mentioned that due this societal stigma, youth with SEMHP are often recognized too late.

##### 4.d Societal stress

Societal stress was described by the participants as systematical societal pressure, affected by (i) a lot of information received by (social) media which may be difficult to process; (ii) usage of social media (presenting a perfect picture of social life); and (iii) environmental problems, such as the climate crisis, and other topics that feel beyond their control. As a result of this societal stress, youth mentioned avoiding strategies, such as staying home sick from school or using drugs.

*“And because of the stress that we systematically place on ourselves and that is also placed on us by society. Then youth show exhibiting behavior quickly, not necessarily deviant, I don't find it so much deviant as socially it is deviant.”* Youth 6

#### Category 5: Impact on daily functioning

A characteristic of youth with SEMHP described by the participants is substantial impact on their daily lives. This impact relates to all contexts including youth as individual, youth’s family, youth’s peers, the mental health care sector and society, and is, therefore, described as a separate category. In this category, we formulated the following themes related to the impact: (a) hampered functioning in multiple life domains; (b) elusiveness of the mental health problems; and (c) deep feelings of hopelessness.

##### 5.a Hampered functioning in multiple life domains

Almost all participants mentioned hampered functioning in multiple life domains associated with youth’s SEMHP. It was described in terms of severe problems in important life domains, namely (i) at school; (ii) at work; (iii) in the family system; (iv) in social relationships; and (v) in the mental health care system. Participants indicated that youth’s problems work both ways: SEMHP affects functioning in various life domains, but are also affected or worsened by the mismatch between multiple life domains. For example, the way the school system is set up may not match youth’s treatment needs (e.g. absence of school due to treatment), which maintained or worsened youth’s problems.

##### 5.b Elusiveness of the mental health problems

Participants described the elusive character of SEMHP as the difficulty to grasp the interrelatedness of youth’s problems. According to some participants, both youth and clinicians, often there is a lot going on simultaneously, making it hard to understand which (underlying) problem is causing which symptoms. As a result, youth often feel misunderstood or experience mistreatment.

*“Suppose you have a form of autism that is not severe. And you also have an eating disorder, which becomes very compulsive. This compulsivity is often associated with an eating disorder. However, in this case, it could be part of autism that plays through. I can imagine that, as a clinician, you think: I don't know anymore.”* Youth 3

##### 5.c Deep feelings of hopelessness in everyone involved

Deep feelings of hopelessness were explained by youth as the lack of hope in everyday life due to (i) a lack of perspective, or nothing to work towards; (ii) a high burden; and (iii) a desperate social environment such as desperate and exhausted caregivers. In addition, clinicians mentioned the importance of paying attention to their own feelings of hopelessness as a result of feeling powerlessness, where as a clinician, you cannot solve everything.

*“Life has been like such a struggle. All kinds of statements have been made about how it could be, but it hasn't been achieved yet. The youth I am talking about also feel and fear the endurance of their problems.”* Clinician 5

## Discussion

This qualitative study provides an in-depth insight into characteristics associated with youth with SEMHP, from the perspectives of youth and clinicians. A first finding was that severity was described in terms of the presence of underlying trauma, problems in multiple life domains, and hospitalization; while enduring was described in terms of the duration of care affecting the duration of the mental health problems. Second, characteristics associated with severe and enduring were beyond individual, and included the environments and systems in which these youth find themselves. This finding is crucial when discussing severity and duration of SEMHP in clinical practice, since the contextual characteristics are merely considered in the current DSM criteria [10]. Third, we consider the recognition of severe and enduring not as a single process, but a long-term cyclic development in which youth’s problems move from a mild–moderate problem into a severe and enduring one. This has vast consequences for clinical practice and assessment. In the following section, we reflect on our key findings and provide recommendations for practice and future research.

### Key finding 1: The interrelatedness of SEMHP in multiple life domains

This study identified characteristics of youth with SEMHP, that not only concern the youth as an individual, but are also related to their families, peers, friends, mental health care (in this case CAP) and society. These different contexts interact and cause mental health problems to be perpetuated or even worsen. It is striking that the problems in different contexts influence each other as vicious cycle, as we too often solely focus on the individual context in CAP. Prior evidence was found for a relation between youth and their contexts, affecting youth’s mental health [35].

In line with the existing literature, we identified the importance of caregivers (micro-system) in the emergence and/or continuation of SEMHP in youth. They may genetically pass on psychiatric vulnerabilities, interpersonal trauma, and affect youth’s functioning due their own stressors [4, 36, 37]. Often, these caregivers’ stressors in combination with youth’s stressors (together familial stressors) results in family conflicts, which are caused by, and causing, deep feelings of hopelessness in both youth and caregivers. Thus, it is not just about the problems of youth or their caregivers, but rather an interactive process over time.

What makes our study unique, is that our results emphasize the importance of understanding the interactive process and the perpetuating effect of familial stressors on youth with SEMHP. This interaction creates a risk for accumulation of complicating factors in other areas of life [38] and should, therefore, be properly assessed in diagnostics of youth with SEMHP in CAP.

Moreover, both this study and previous research found that youth with SEMHP regularly drop out of school and show problems in peer relationships and family life [2, 4, 39]. Our study exposed potential reasons why these problems arise, such as the lack of social support by peers and family members. In line with the existing literature [40], we identified that youth with SEMHP often experience negative reactions or distance from their peers due to stigma and lack of knowledge about their problems, resulting in school difficulties. It is therefore of upmost importance to also support youth with SEMHP outside of CAP, and in other life domains such as school. A recommendation would be to strengthen the social network of youth with SEMHP (and their family), for example with a mentor from school and/or a close friend, so that youth (and their family) receives adequate support [41, 42].

However, many schools lack the knowledge and resources necessary to support these youth [43, 44], Support in education must be better aligned with the deployment from the youth care system [45, 46]. In the Netherlands, the Care Advisory Team (CAT) is an example of good collaboration between education and mental health care [45]. Such multidisciplinary teams can quickly assess early signals of SEMHP from teachers that indicate youth’s needs for support [45].

### Key finding 2: The long-term nature of SEMHP and its hiddenness

Severe and enduring mental health problems are not always visible for youth themselves and their surroundings, including their caregivers, peers and clinicians. This hiddenness can be partly explained by the gradual onset of the mental health problems during the development of youth and youth’s late use of mental health services [47]. On top of that, this study identified characteristics of youth with SEMHP that also contribute to its hiddenness, namely youth masking their problems, and interpersonal distrust in themselves. We discovered that youth with SEMHP tend to mask their emotions to unburden their caregivers, who often experience (personal) stressors themselves. In addition, youth mask their problems to fit in with peers due to a need to belong [48]. In line with the existing literature [36, 40, 49], we identified that youth often fear judgement or misunderstanding from their peers, family, and society.

Moreover, masking or hiding mental health problems in life and in treatment can lead to high-risk behavior, such as deliberate self-harm, suicidal behavior, and disordered eating as these youth may seek to alleviate their distress [50]. Similar to the interaction of familial stressors, this seems to be an interactive process in which youth become increasingly stuck in their mental health problems and, therefore, mask them more. These behaviors can be seen as avoidant behavior, which is easily misinterpreted as a lack of engagement by clinicians [51] or a rebellious or aggressive attitude [52], while in reality, it may be a manifestation of their underlying mental health difficulties. Therefore, it is important for clinicians, peers, and caregivers to be aware of the potential misinterpretation of masking and high risk behavior as demotivated, disengaged, rebellious or an aggressive attitude. In that, they should attempt to discover the underlying explanations driving this behavior which (mental health) problems being masked or avoided and for what reasons.

### Key finding 3: Potential indicators of deficits for youth with SEMHP in current systems

This study identified multiple potential indicators of deficits within the mental health care system and society for youth with SEMHP. First, the presence of multiple classifications and hospitalization were associated with SEMHP, indicating that youth’s care history is important to take into account. In line with the existing critics on the DSM-5 [7, 53], youth with SEMHP are often over-diagnosed by multiple classifications. According to our findings, this is because all problems are seen separately and, therefore, the interrelatedness of problems is overlooked.

Moreover, emergency clinical admission is a common intervention for this SEMHP group, often resulting in hospitalization after an extended period of time [54]. While a prior study underlined the improvement in functioning after hospitalization [55], the participants in our study described that life within care can be detrimental to youth’s mental health and well-being, leading to the disappearance of a future perspective on daily life [56]. Secondly, youth living in the current society often experience stress caused by social media. This potentially exposures negative or self-harm related content, worsening negative feelings in youth with SEMHP [57].

Hence, it is important for clinicians, policy makers, youth, peers, and caregivers to be aware of the potential indicators of deficits within the mental healthcare system and society, contributing to the severity and duration of youth’s mental health problems. The mental health care system (including policy makers) need to be critical of the role of classifications in the available treatment options and strive to a more person centered approach [58]. Also, the novelty of this study is that our results underline the importance to consider the cultural and societal expectations [59] and stressors that come along with growing up in the twenty-first century, such as a negative social media effect [60], in the understanding of SEMHP in youth.

### Strengths and limitations

A strength of this study is the explorative qualitative nature, which is a valuable approach to understanding the characteristics of youth with SEMHP in a more context-dependent, interactive, and nuanced manner [61]. However, a qualitative approach does not lend itself to generalizing the characteristics [61]. Rather our results are transferable, since we reported descriptive information about the research setting, our participants and our processes [61, 62] according to the COREQ guidelines [21]. Our purposive sampling strategy was fitting to include youth with lived experience and specialized clinicians, because of their experiences with severe and enduring mental health problems. However, we are aware that the relatively small group of youth (*n = *10) who participated in this study does not represent the whole target group. Further research is warranted to explore whether the identified characteristics are discernible among a broader population in CAP.

Also, performing a thematic analysis could potentially be biased, since interpretations and conclusion can be influenced by personal experience and knowledge [63, 64]. An effort to overcome such bias, were our reflective meetings to discuss the identified themes [65].

Other strengths of this study include timing of data collection during COVID-19 pandemic (a unique context) and findings on social media and uncertainties about future related to climate and economic changes. New insights into severe and enduring mental health problems in current time helps us to gain a better understanding of what our target population (new generation) needs.

In addition, this study focused on youth with SEMHP in the context of CAP facilities. We acknowledge that there are youth with SEMHP who are treated outside this setting and youth who are not in care at all for many different reasons [4].

This study is the first to qualitatively explore the characteristics of youth with SEMHP from the perspectives of youth with lived experience and specialized clinicians. We aimed at exploring what characteristics were described as important by both participant groups. By incorporating these perspectives, which is crucial for providing valuable insights into the experiences and needs, clinical practice and research can gain a better understanding of youth with SEMHP and enhance care for these youth. For a follow-up study, it would be valuable to choose a design that better lends itself to compare the perspectives of both youth and clinicians. By, for example, administering Likert-scale questionnaires to a larger group of participants, it would be possible to explore potential differences or similarities in perspectives. Also, not including caregivers’ perspectives may have limited the scope of this study and overlooked important insights into the characteristics of these youth. This is because of crucial involvement of caregivers in the lives of these youth. For this reason, future research should include the perspective of caregivers.

## Conclusion

This study identified multiple characteristics associated with SEMHP by youth and clinicians, which are not only individual, but also concern the environments and systems in which these youth find themselves. Therefore, we recommend proper assessment of the characteristics in all life domains (home, school, mental health care and society) affected and their perpetuating effect on SEMHP during diagnostics in CAP. It is highly important to engage in conversation with youth themselves, due to the nature of their characteristics, which frequently transcend traditional classifications and may not be immediately discernible. It also requires an integrated care approach, entailing collaborations between educational institutions and mental healthcare providers, and attention to potential indicators of deficits in the health care system and society.

### Supplementary Information

Below is the link to the electronic supplementary material.

Supplementary file1 (PDF 419 kb)

Supplementary file2 (DOCX 15 kb)

Supplementary file3 (DOCX 14 kb)

Supplementary file4 (DOCX 16 kb)

Supplementary file5 (DOCX 18 kb)

## Data Availability

The datasets generated and analyzed during the current study are available from the corresponding author on reasonable request.
