# Supplementary material for: Towards identifying the characteristics of youth with severe and enduring mental health problems in practice: a qualitative study
Source: Eur Child Adolesc Psychiatry. 2023 Dec 26;33(7):2365–75. doi: 10.1007/s00787-023-02325-2 (PMC11255042; doi:10.1007/s00787-023-02325-2)
Supplement: Supplementary file 2 — Supplementary file2 (DOCX 15 kb) [file 787_2023_2325_MOESM2_ESM.docx]

**Appendix B. Topic-list youth with lived experience and specialized clinicians**

| **Topics** | **Questions** |
| --- | --- |
| Motivation for participating | - What was your reason for participating in this interview? |
| Meaning severe and enduring mental health problems | - We will explore a target group described as youth with severe and enduring mental health problems: what is the first thing that comes to mind when you think of this? |
| Enduring mental health problems | - How would you describe enduring mental health problems? |
| Recognition of enduring mental health problems | - How can one recognize that mental health problems are or are becoming enduring? What are the signals? - Do you think the environment (network/school) recognizes these signals? - Do you think youth themselves recognize these signals? - How could mental health care recognize these signals more early? - What factors contribute to enduring mental health problems? |
| Severe mental health problems | - How would you describe severe mental health problems? |
| Recognition of severe mental health problems | - How can one recognize that mental health problems are becoming severe? What are the signals? - Do you think the environment (network/school) recognizes these signals? - Do you think youth themselves recognize these signals? - How could mental health care recognize these signals more early? - What factors contribute to severe mental health problems? |
| Visibility to the mental health care system | - Do you feel these youth are visible to the mental health care system? Why yes/no? |
| Societal change | - Is there anything in society you would like to change for youth with SEMHP? If so, what and why? |
| Closing questions | - What did you think of the interview? - Are you satisfied with the things you said? - Are there any things you would like to say that I did not ask? - Finally, do you have any questions? |
