# Supplementary material for: Towards identifying the characteristics of youth with severe and enduring mental health problems in practice: a qualitative study
Source: Eur Child Adolesc Psychiatry. 2023 Dec 26;33(7):2365–75. doi: 10.1007/s00787-023-02325-2 (PMC11255042; doi:10.1007/s00787-023-02325-2)
Supplement: Supplementary file 3 — Supplementary file3 (DOCX 14 kb) [file 787_2023_2325_MOESM3_ESM.docx]

**Appendix C. Coding framework**

|  | Codes from the coding scheme | Frequency of quotes per code |
| --- | --- | --- |
| 1. Framework (literature-based) | Severe  Enduring | 167  207  92  169 |
|  | Risk factors  Indicators |  |
|  | Impact | 57 |
| 2. Open coding | Diagnoses  Age | 98  33 |
|  | Vulnerable  Network  Perspective | 25  111  31 |
|  | Hampered functioning | 51 |
|  | Safety | 26 |
|  | Changing mental health problems | 29 |
|  | Avoidance | 26 |
|  | Trust | 54 |
|  | Motivation | 28 |
|  | Powerlessness | 45 |
|  | Despair | 32 |
| 3. Overarching coding | Concepts  Personal Characteristics  Environmental Characteristics | 167  98  57 |
