# Supplementary material for: Towards identifying the characteristics of youth with severe and enduring mental health problems in practice: a qualitative study
Source: Eur Child Adolesc Psychiatry. 2023 Dec 26;33(7):2365–75. doi: 10.1007/s00787-023-02325-2 (PMC11255042; doi:10.1007/s00787-023-02325-2)
Supplement: Supplementary file 4 — Supplementary file4 (DOCX 16 kb) [file 787_2023_2325_MOESM4_ESM.docx]

**Appendix D. Descriptions of enduring and severe**

| **ENDURING** | **Theme (mentioned by**  **n =*participants)** | **Description** |
| --- | --- | --- |
|  | Duration of mental health problems  (n = 5) | The duration of mental health problems ranged from a minimum of six months (one clinician) to more than two years (two clinicians and two youth). |
|  | Duration of care  (n = 9) | Youth (n = 5) described enduring in terms of the duration of receiving mental health care (i.e., length of treatment) and not receiving care (i.e., constant referrals and waiting lists). |
|  | Recurrency of the problems  (n = 5) | Youth (n = 3) and clinicians (n = 2) described the nature of enduring problems as alternating problems, in which mental health problems appear recurrently. |
|  | The invisibility of the problems  (n = 5) | Youth (n = 5) mentioned that some of the mental health problems were hard to see for a long time, and therefore become ‘invisible’ for themselves and their environment. |

| **SEVERE** | **Theme (mentioned by**  **n =*participants)** | **Description** |
| --- | --- | --- |
|  | Hampered functioning on various life domains (n = 15) | Severity was mainly described by youth (n = 10) and clinicians (n = 5) as being stuck, a significant hampering in functioning on multiple life domains (e.g., school absence or a loss of social relationships). |
|  | Trauma (n = 13) | Underlying trauma (mainly due to abuse during their childhood) was described by youth (n = 4) and clinicians (n = 5) as an essential part of severe mental health problems. |
|  | Multiple classifications  (n = 9) | Both youth (n = 5) and clinicians (n = 4) mentioned multiple mental health problems being severe. They described receiving multiple classifications as affecting the treatment outcome. |
|  | Hospitalization  (n = 7) | The impact of clinical admissions on the severity of their mental health problems was also described. According to youth (n = 5), severity can increase due to hospitalization and isolation from society. |
|  | High-risk behavior (n = 8) | Both youth (n = 3) and clinicians (n = 5) speak of severity in relation to high-risk behavior and safety issues, including suicidality, self-mutilation, aggression, eating problems, or unsafety behavior towards the environment. |
|  | High burden  (n = 4) | Participants also described severity as suffering, despair, and low quality of life. |
