# Supplementary material for: Towards identifying the characteristics of youth with severe and enduring mental health problems in practice: a qualitative study
Source: Eur Child Adolesc Psychiatry. 2023 Dec 26;33(7):2365–75. doi: 10.1007/s00787-023-02325-2 (PMC11255042; doi:10.1007/s00787-023-02325-2)
Supplement: Supplementary file 5 — Supplementary file5 (DOCX 18 kb) [file 787_2023_2325_MOESM5_ESM.docx]

**Appendix E. Frequency of characteristics associated with SEMHP**

| **Category** | **Description** | **Themes** | **Frequency of themes per # participants**  (Youth / Clinicians) |
| --- | --- | --- | --- |
| **Individual context** | | |  |
|  | This category describes characteristics of youth with SEMHP related to individual factors. | Trauma  Genetic vulnerability  Puberty  Masking  Self-destructive behavior  Interpersonal distrust | Y: 7 / Cl: 6  Y: 0 / Cl: 6  Y: 3 / Cl: 3  Y: 3 / Cl: 4  Y: 2 / Cl: 4  Y: 6 / Cl: 3 |
| **Family context** | | |  |
|  | This category describes characteristics of youth with SEMHP related to family factors. | Parental stress  Parental psychiatric problems  Parental financial problems  Communication | Y: 6 / Cl: 6  Y: 2 / Cl: 4  Y: 0 / Cl: 2  Y: 0 / Cl: 1 |
| **Peer context** |  |  |  |
|  | This category describes characteristics of youth with SEMHP related to peer factors. | Social network  Lack of support  Isolation  Invisibility | Y: 9 / Cl: 7  Y: 5 / Cl: 1  Y: 5 / Cl: 1  Y: 3 / Cl: 6 |
| **Societal context** |  |  |  |
|  | This category describes characteristics of youth with SEMHP related to factors in the mental health care system and society. | Mental health care system  Multiple classifications  Hospitalization  Society  Stigma  Stress | Y: 3 / Cl: 3  Y: 5 / Cl: 4  Y: 4 / Cl: 2  Y: 4 / Cl: 4  Y: 4 / Cl: 1  Y: 2 / Cl: 1 |
| **Impact** |  |  |  |
|  | This category describes characteristics of youth with SEMHP related to the impact on their daily lives. | Stagnation in multiple life domains  Elusiveness  Hopelessness | Y: 10 / Cl: 9  Y: 5 / Cl: 2  Y: 5 / Cl: 6 |
